# From responsibility to responsibilization in stem cell research: An ethical framework

**DOI:** 10.1016/j.stemcr.2024.102389

**Published:** 2025-01-09

**Authors:** Lars S. Assen, Annelien L. Bredenoord, Rosario Isasi, Morris A. Fabbri, Marianna A. Tryfonidou, Karin R. Jongsma

**Affiliations:** 1 Radboud University Medical Centre, Department of IQ Health, Kapittelweg 54, 6525 EP Nijmegen, the Netherlands; 2 Erasmus School of Philosophy, Erasmus University Rotterdam, 3000 DR Rotterdam, the Netherlands; 3 Department of Human Genetics & Interdisciplinary Stem Cell Institute, Dr. John T. Macdonald Foundation, University of Miami Miller School of Medicine, Miami, FL 33136, USA; 4 Department of Clinical Sciences, Faculty of Veterinary Medicine, Utrecht University, 3584 CM Utrecht, the Netherlands; 5 Department of Bioethics and Health Humanities, Julius Center for Health Sciences & Primary Care, University Medical Center Utrecht, PO Box 8500, 3508 GA Utrecht, the Netherlands

## Abstract

Adopting a responsibilization approach can further improve the ethical conduct of stem cell (SC) research and applications. This approach helps align new and existing solutions for ethical implications by focusing on equipping SC researchers with the knowledge, skills, and organizational arrangements to take (co-)responsibility for the socio-ethical implications of their research.

## Introduction

The history of stem cell (SC) research and its promises is paired with socio-ethical implications. As such, decades’ worth of ethical research has analyzed issues surrounding the (embryonic) sourcing of human pluripotent SCs, the ethical requirements for use in research, the potential health risks of SC interventions, as well as responsibilities regarding science communication and public engagement (de Wert and Mummery, 2003; Sugarman et al., 2023; Assen et al., 2021). Moreover, the clinical translation of SC research and its regulation, the dangers of SC tourism, and the moral status of SC-based applications have also widely been discussed (Chan, 2017; Macpherson and Kimmelman, 2019).

The ethical conduct of SC research and applications can further be improved by adopting a more comprehensive framework that synthesizes new and identified ethical challenges with existing and new solutions. Such an approach helps to clarify how responsibilities are distributed and which knowledge, skills, and appropriate organizational and regulatory arrangements are needed (Illes et al., 2017; Mejlgaard et al., 2020; Haven et al., 2020; Assen et al., 2022).

To accommodate this, we provide a framework for responsibilization (Table 1), which equips researchers in recognizing and taking responsibility for the ethical implications of their research. In our framework, we aim to equip SC researchers on multiple levels: ranging from individual training in knowledge and skills to organizational and regulatory changes. We identify target areas of responsibilization by discussing existing ethical challenges and propose advances for the responsible conduct of SC research and dealing with other ethical implications of SC research. As the field is in constant development, this framework can only be considered as a starting point to foster responsibilization in SC research.

**Table 1:** Framework for responsibilization

Challenges		
Governance	Responsible research conduct in a rapidly evolving context	SC Science in and with Society
1. Uncertainty whether governance frameworks are fulfilling their stewardship roles due to outdated guiding norms 2. Governance frameworks unresponsive to changing research practices 3. Mere compliance could neglect ethical reflection on the research practice by SC researchers	4. Lack of knowledge of new technologies could limit the quality of supervision and research integrity 5. Unintended and unforeseen effects of science and technology during research 6. Lack of replication studies due to unclear or faulty methodology sections and lack of funding 7. False expectations and SC hype 8. Possible lapses in research integrity	9. Societal priorities, expectations, and societal values are not always clear or included in research 10. Realizing meaningful societal and patient participation 11. Public engagement does not always lead to a positive experience for (early career) researchers 12. Anticipating the effects of SC research and technology on social structures, moral values, and behavior

Solutions		
Responsible conduct and innovation	Educating and training for responsible innovation	Sharing and disseminating
a. Fostering a research integrity climate following open science initiative; implementing fair assessment procedures; qualitative supervision and role models (4, 8) b. Fostering transparency toward the public and academia (7, 8, 10) c. Introducing and adopting clear reporting guidelines for methodology sections and registering prospective trials (6, 8) d. Initiating research to establish whether regulation is still fulfilling its stewardship role (1, 2) e. Raising additional funding for replication studies and rewarding researchers for conducting replications studies (6, 8) f. Implementing sessions in research groups to reflect on the broad ethical implications of SC research (2, 3, 12) g. Realizing administrative support for regulatory affairs and ethics applications (3, 8) h. Rewarding researchers for anticipating the ethical implications of their research (2, 3, 8)	i. Educating in rules and regulation (8) j. Virtue-based responsible conduct of research training leading to less scientific misconduct, more compliance in general and promote moral reflection (2, 4, 8) k. Training in moral reflection through moral imagination and hard and soft impacts (2, 5, 12) l. Training researchers for responsible innovation to be adaptive to changes and to understand how responsibilities are shared and distributed (2, 3, 4, 5, 8) m. Train researchers in structured and timely reporting (7, 9)	n. Educating researchers about dissemination strategies (9, 10, 11) o. Training researchers in using non-scientific language, storytelling, and translating societal questions to research questions (8, 10) p. Establishing Patient/Community Advisory Boards in research consortia for science communication (9, 10, 12) q. Rewarding researchers for patient and public involvement and for integrating patient and public perspectives in research (9, 10, 11, 12) r. Realizing public deliberation activities for insights in societal values and priorities. Subsequently, aligning research goals with these societal values and priorities (9, 10, 12) s. Implementing science communication offices that support researchers in dissemination activities (10, 11)

The numbers in brackets following the solutions correspond to the respective challenges.

## What responsibilization should target?

### Governance

Governance is defined as a process by which authority is administered and oversight is exercised through rules (e.g., standards of practice, codes of conduct, legislation, and professional guidelines) and the process by which actors (e.g., individuals, institutions, and communities) are held accountable. Various governance frameworks are in place to ethically and legally guide SC research. Yet, in some instances, these frameworks pose problems for responsible conduct and innovation, for example, when the guiding norms are outdated and therefore do not sufficiently steward SC research (Isasi et al., 2022) or when such governance frameworks are not responsive to changing research practices or societal attitudes. Furthermore, ethical reflection is hampered when ethical aspects are perceived by researchers as a tick-the-box-exercise (Assen et al., 2022), or when focusing solely on compliance with legislation and policy (Assen et al., 2022) rather than on substantially addressing key socio-ethical issues.

Additionally, policy, legislation, and education aim to guide ethical research behavior in SC research. Still, research integrity concerns persist in this field, indicating that existing policy and educational efforts to increase needed knowledge and skills of individual researchers do not suffice to tackle organizational and structural factors that are known to contribute to lapses in research integrity (Mejlgaard et al., 2020; Haven et al., 2020).

### Responsible research conduct in a fastly evolving context

Responsible research conduct with new technologies and methodologies is challenging. For example, due to technological and methodological advancements in the field, late-career researchers may be insufficiently up to date with new techniques and practices to adequately supervise their early-career counterparts (Assen et al., 2022). Likewise, researchers may face scientific challenges, as, for instance, lacking understanding regarding dataset limitations because of unfamiliarity, thus hindering the quality of research (Sand et al., 2022). For example, artificial intelligence and big-data research are becoming more integrated in the SC field, but it is unclear whether researchers understand sufficiently how artificial intelligence technology affects their research from an ethical perspective.

Scientific progress is enabled by the transparency of findings and funding, among other factors. However, transparency is at risk due to unclear methodology sections in scientific publications and poor metadata management. When peers cannot obtain comprehensive information about the research methods, replication studies are not feasible. In turn, this could prevent scientific validation and hamper scientific progress. Similarly, information regarding funding sources is important to contextualize findings and distill potential conflict of interests or biases.

### SC science in and with society

Interactions with the public and patients are considered important to contribute to trust in research and realize societally relevant research (Boon et al., 2022). This is particularly important for the SC field, where public engagement activities can provide insights into research priorities, align with societal needs, and promote wider acceptance of SC-based therapies by society. To realize more engagement also in earlier phases of research, researchers, especially early-career researchers, should be supported to discuss ethically sensitive aspects of SC research. Additionally, inaccurate media coverage should be countered in order to promote realistic societal expectations (Assen et al., 2022). Furthermore, aligning SC research with societal values has gained increased relevance. One example concerns ecological sustainability, as biomedical laboratories often have a high energy consumption and usage of plastic disposable items (Banks et al., 2020). It has been recognized that different materials and new ways of working could reduce the carbon footprint, such as opting for glass or autoclavable plastics and implementing protocols and procedures to repurpose or minimize plastic use (Banks et al., 2020).

## Advancing responsibilization

### Responsible conduct and innovation

Responsible conduct of research and research integrity could be further improved, for instance, by operationalizing and following recommendations of the International Society for Stem Cell Research (ISSCR). These recommendations focus on fostering integrity by calling for transparency about successful and unsuccessful experiments and in the communication of the origin of SC, among other goals. Additionally, to foster medical and scientific merits, the ISSCR recommends to publicly register prospective trials. In addition, the UK Academy of Medical Sciences proposed best practices to further advance responsible research conduct. These best practices include promoting post-publication reviews, publishing study protocols, and promoting open data by openly sharing results and underlying data. Moreover, the increased attention of research funders for the reproduction of research studies is a promising avenue to further the SC field; additionally, clearer reporting guidelines for methodology sections, as also suggested by Academy of Medical Sciences, would further strengthen the practice of responsible innovation.

On an organizational level, research institutes can further contribute to an environment that stimulates research integrity. Best practices already exist to implement assessment procedures and reduce academic carreer pressure. Such assessment procedures include—among others—peer judgment of the quality of research, public engagement, and educational activities rather than assessments based on h-indices and publication records (Mejlgaard et al., 2020), which have been adopted by the coalition of advancing research assessment and individual universities. In addition, positive role models could contribute to creating an environment that promotes research integrity (Haven et al., 2020). Research integrity could also be promoted by unburdening researchers and reducing work load through practical and administrative support for regulatory and ethics applications (Haven et al., 2020). Furthermore, additional research is required to understand whether contemporary regulation and policy fulfill a stewarding role for the conduct of SC research (Isasi et al., 2022).

To consider the broader ethical challenges of SC research, research institutes and individual research groups could implement sessions with fellow SC researchers to consider how the indirect effects of SC research and technology could affect social structures, moral values, psychology, and behavior—the so-called soft impacts (Assen et al., 2021).

### Education and training for responsible innovation

Educating and training researchers can help to promote responsible research and innovation. For example, innovation is often paired with new risks, possible benefits, and new responsibilities. To anticipate these new responsibilities, education should focus on the limitations of new technologies and the competencies required to deal with these technologies responsibly (Sand et al., 2022). To better recognize the broad scope of ethical implications, researchers and students could be trained in moral reflection on the implications of SC research, such as educating them about the soft impacts and by means of moral imagination (Assen et al., 2021). Additionally, virtue ethical approaches seem a valuable way to promote responsible research conduct by equipping researchers with the necessary knowledge, skills, and attitudes (Goddiksen and Gjerris, 2022). Furthermore, such an approach clarifies the position of researchers within their research group, institution, and the field of SC research (Goddiksen and Gjerris, 2022), providing researchers understanding of how responsibilities are shared and distributed in their organization and in the broader SC field.

### Sharing and disseminating

Inclusion of patients and the public in dissemination of SC research is important for fostering both responsible conduct and responsible innovation. Science communication with patients and the public can be promoted by improving the knowledge and skills necessary for dissemination of SC researchers. For instance, training and education of researchers on how and when to involve the public and patients in research (Assen et al., 2022) could foster the understanding of public perspectives. When dissemination is approached from the deficit model, where scientists assume the public lacks knowledge, it leads to limited interaction and understanding of each other. Instead, a dialogue model offers room for acknowledging different perspectives (Assen et al., 2022). To foster this, researchers need to be further trained in skills to communicate in accessible language, in listening, in storytelling, and in translating societal questions to research questions and research questions to societal questions (Boon et al., 2022).

On an organizational level, the latter can be facilitated by science communication officers that support individual researchers for both written and spoken dissemination activities (Assen et al., 2022). Recognition and rewards programs could acknowledge researchers realizing patient and public involvement and integrating public perspectives in their research. Furthermore, public and patient perspectives could be included by ensuring that guidelines, like the ISSCR’s, reflect societal values and priorities. Those values and priorities could be specified by realizing public deliberation activities (Isasi et al., 2022).

Facilitating Patient Advisory Boards can help for effective dissemination. For instance, it is an increasingly common practice to implement Patient Advisory Boards in national and international research consortia for better inclusion of patient perspectives and effective science communication (Anderson and Getz, 2018). Altogether, such actions can promote the valuable inclusion of the patients and the public in SC research.

## Discussion

This article offers a comprehensive framework for responsibilization to promote the responsible conduct and innovation in SC research. It contributes to the existing literature by combining the identified ethical challenges with possible solutions and outlining how individual stakeholders can be equipped to take responsibility.

To this end, three dimensions of possible ethical challenges have been identified: *governance*, *responsible research and innovation in a rapidly changing environment*, and *stem cell research in and with society*. The solutions addressing these challenges concern three areas: *responsible conduct and innovation*, *educating and training for responsible innovation*, and *sharing and disseminating*. Within these areas, considerations on multiple levels are needed to enable the responsibilization of SC researchers: from organizational arrangements that support researchers in knowledge distribution, to collaboration and sharing responsibilities, to improving skills in dealing with the ethical implications of SC research. As a result, the framework has several merits.

First, the framework focuses on whether and how individual stakeholders, such as SC researchers, can uphold responsibility. This differs from efforts that primarily focus on ethical challenges, responsibilities, or specific goals (Chan 2017; Macpherson and Kimmelman, 2019; Sugarman et al., 2023). In essence, the framework defines a line of action to equip researchers with skills, knowledge, and support to take responsibility for their professional actions. While doing so, the described framework pays attention to organizational and structural changes that could alleviate the workload of individual SC researchers and reduce incentives that might stimulate unethical behavior.

Second, the framework draws the attention to the contexts in which responsibilities are embedded by creating an overview of the distribution of responsibilities and defining how certain responsibilities are interconnected. For example, it has been argued that successful public engagement should focus on realizing seven goals: “(1) avoid potential controversy, (2) educate the public, (3) build democratic capacity through deliberation, (4) widen representation of voices, (5) solicit input on value debates, (6) enable responsible innovation, and (7) shape policy” (Sugarman et al., 2023, p. 423). The question that remains is how these goals can be achieved. To that end, the perspective of responsibilization emphasizes considerations of what SC researchers need to realize those goals, such as training or support for transferring knowledge, developing specific communication skills, and organizing opportunities for researchers to engage with the public. Accordingly, it becomes clearer which actors could play a role and share responsibilities to realize successful public engagement through training and support. As a result, a more comprehensive understanding of the possible role of individuals and organizations can be created, and specific responsibilities and expectations can be formulated. In turn, this could help to fill or prevent possible gaps in responsibility.

Finally, our analysis should be considered as a precursor for a comprehensive framework on responsibilization that does not necessarily apply only to SC research but also to other disciplines in the field of biomedical and regenerative medicine research. As such, it is open to include existing and new identified ethical challenges and solutions to ethical challenges. We invite other scholars to elaborate or revise this framework with the intention to further the field of SC research toward responsible innovation and suggest our solutions to ethical challenges as recommendations for the field of SC research.

## Acknowledgments

We would like to thank the researchers and the PAB of the iPSpine consortium for their constructive input. This project has received funding from the European Union’s Horizon 2020 research and innovation program iPSpine under grant agreement no. 825925. M.A.T. receives funding from the 10.13039/100018286Dutch Arthritis Society (LLP22).

## Author contributions

L.S.A., K.R.J., and A.L.B. prepared the initial draft. M.A.T., R.I., and M.A.F. revised several versions of the manuscript for intellectual content. All authors read, contributed to, and approved the final version.

## Declaration of interests

The authors declare no competing interests.
